# Mapping Perceived Partner Phubbing, Triangular Love, and Relationship Quality: A Psychometric Network Analysis

**DOI:** 10.3390/bs16101810

**Published:** 2026-10-03

**Authors:** Müzeyyen Soyer, Seçil Ömür Sünbül

**Affiliations:** Department of Educational Sciences, Mersin University, Mersin 33343, Türkiye; secilomur@gmail.com

**Keywords:** perceived partner phubbing, triangular theory of love, network analysis, bridge centrality, intimacy, passion, commitment, relationship quality

## Abstract

Partner phubbing—attending to one’s phone during interactions with a romantic partner—has been associated with poorer relationship functioning, yet its behavior-level links with the components of Sternberg’s triangular theory of love remain unclear, and existing evidence is largely variable-centered. This study estimated a psychometric network linking specific perceived partner phubbing behaviors with intimacy, passion, commitment, and global relationship quality. A cross-sectional study was conducted with 476 adults in romantic relationships. Partner phubbing, triangular love, and perceived relationship quality were assessed using validated questionnaires. A regularized partial-correlation network (EBICglasso based on Spearman correlations) was estimated across 13 nodes. Two coherent communities emerged, representing phubbing and relationship constructs. Within-community connections were strong and positive, whereas cross-community connections were negative and very weak; no cross-community partial correlation exceeded |0.05| despite small-to-moderate zero-order correlations (ρ = −0.25 to −0.33). Global relationship quality and intimacy showed the strongest links to phubbing, whereas passion and commitment were more peripheral. Behaviors involving phone use during interaction (glancing at the phone while talking, checking it mid-conversation, and using it while out together) showed relatively higher estimated cross-community associations, although these were weak and not individually reliable. Expected-influence centrality was highly stable (CS = 0.75), bridge centrality showed moderate stability (CS = 0.45), and findings were robust across correlation methods (edge-weight r = 0.98). Overall, partner phubbing and triangular love formed two coherent but weakly connected communities. The observed behavior-level bridges should be interpreted as hypothesis-generating rather than evidence of causal relationships. Because phubbing was reported by only one partner, the findings concern perceived partner phubbing rather than directly observed behavior and may be affected by single-informant bias.

## 1. Introduction

Mobile phones now accompany most couples through the ordinary hours of the day. They sustain contact when partners are apart, yet the same devices enter the moments couples reserve for one another. Researchers describe these everyday intrusions as technoference (McDaniel & Coyne, 2016); their most visible form in dyadic interaction is partner phubbing—attending to a phone while a romantic partner is present (Chotpitayasunondh & Douglas, 2016; Roberts & David, 2016). A single episode is brief and easily excused, yet such interruptions are common in couple interaction across countries (Karadağ et al., 2015; McDaniel & Coyne, 2016). The behavior is therefore easy to dismiss one instance at a time and difficult to dismiss in aggregate.

Frequency alone would not warrant attention. What gives phubbing its research interest is its repeated association with poorer relational outcomes in the literature that has grown quickly (Ni et al., 2025). Cross-sectional work links perceived partner phubbing to lower relationship satisfaction and to conflict over device use (McDaniel & Coyne, 2016; Roberts & David, 2016), and comparable associations appear with lower intimacy and poorer relationship quality (Beukeboom & Pollmann, 2021; Yam, 2023) and with depressive symptoms among married adults, for whom relationship satisfaction accounts for part of the association (Wang et al., 2017). A cross-lagged study suggests that device use during shared time precedes later declines in satisfaction (Halpern & Katz, 2017), and a recent meta-analysis reports associations that are consistent in direction but modest in magnitude (Ni et al., 2025). Partner phubbing thus appears to be neither trivial nor dramatic, but a small and persistent strain.

Why a brief glance at a screen should matter concerns attention and its social meaning. Partners signal availability through attentiveness, and turning to a phone can be read as a violation of what a partner expects at that moment (Kelly et al., 2017). Experimental evidence indicates that being phubbed lowers perceived communication quality and threatens basic social needs (Chotpitayasunondh & Douglas, 2018). Interpretation matters as much as the act itself, since the same behavior may be excused or resented depending on how it is understood (Beukeboom & Pollmann, 2021), and repetition adds a further layer, because phubbing tends to become normalized and reciprocated rather than challenged (Chotpitayasunondh & Douglas, 2016). The same behavior can also arise for very different reasons, and this boundary is easily blurred: turning to a phone out of indifference is not the same as using it to manage sensory load, sustain attention, or process information, a regulatory use of technology that is well documented (Wadley et al., 2020) and that may be especially relevant for some autistic or ADHD individuals. Reading such device use as a lack of affection would misread that experience, so the meaning a partner assigns to phubbing, rather than the behavior alone, is central. The distinction matters. Withdrawing to a device out of indifference, or as a way of rejecting the interaction, is not the same as using it to ease sensory hypersensitivity, support working memory, or accommodate multitasking, which is a form of adaptive self-regulation (Kapp et al., 2019). Treating a single neurotypical standard—such as constant eye contact, or keeping the phone face-down during conversation, as the only valid expression of affection—can itself act as a discriminatory boundary (Milton, 2012) that misclassifies these adaptive behaviors as neglect. On this account, the relational cost of phubbing may lie less in the device than in the withdrawal of attention it enacts. This attention-based account is not the only possibility. A negative association between perceived phubbing and relationship evaluations could also arise if less satisfied partners perceive and report more phubbing, if both reflect general conflict, problematic smartphone use, insecure attachment, or jealousy, or if couples differ in shared norms about device use (McDaniel & Coyne, 2016; Ni et al., 2025). A cross-sectional, undirected network estimates conditional associations at a single time point and cannot distinguish these accounts or establish direction, so we treat the attention-based reading as one interpretation among several.

If phubbing operates through attention and responsiveness, its association with love should not be uniform. Sternberg’s triangular theory describes love as a combination of intimacy (closeness, warmth, and disclosure), passion (attraction and desire), and commitment (the decision to maintain the relationship over time) (Sternberg, 1986, 1997). The three components develop through partly different processes and may therefore differ in their sensitivity to daily interaction. Intimacy is sustained by moment-to-moment responsiveness and shared attention, so it is the component most closely associated with what happens during joint activity. Passion is driven by attraction and arousal, and commitment reflects a longer-term decision; both depend less on any single interaction and more on processes that accumulate over time. Because phubbing occurs in shared, face-to-face moments, it should be tied most closely to intimacy and to the global appraisal partners form of their relationship (Fletcher et al., 2000), whereas links with passion and commitment may be weaker, since both rest on processes extending well beyond momentary attention. Because phubbing during shared moments may withdraw precisely this attention and responsiveness, it is expected to bear more on intimacy and on the global evaluation of the relationship than on passion or commitment (Kelly et al., 2017; Roberts & David, 2016). Global relationship quality is treated here as an overall evaluation of the relationship rather than as a component of love; it is expected to pattern closely with intimacy because both reflect how well the relationship is seen to be going. This expectation has not been tested directly, because studies typically relate phubbing to one global outcome rather than to the separate components.

That observation leads to a methodological point. Research on partner phubbing has relied mainly on total scores, bivariate correlations, and regression or mediation models (Ni et al., 2025; Yam, 2023). Such designs establish whether phubbing is associated with an outcome, but they cannot show which behaviors are most closely associated with romantic experience, or which facets of love are most strongly associated with it, once all are considered together; summing nine behaviors also assumes that reaching for the phone during a meal and glancing at it mid-sentence carry similar relational weight, an assumption that is plausible but untested. Psychometric network analysis offers a way to relax it. Each behavior and construct is treated as a node, and each edge is a conditional association that remains after adjustment for all other nodes (Borsboom & Cramer, 2013; Epskamp & Fried, 2018). Centrality indices identify the most connected nodes (Robinaugh et al., 2016), bridge indices identify nodes that link otherwise separable groups (Jones et al., 2021), and predictability indicates how much of each node’s variance its neighbors explain (Haslbeck & Waldorp, 2018). Throughout, the phubbing nodes represent one partner’s perception of the other’s phone use, measured by a self-report scale, rather than directly observed conduct; they are therefore best understood as perceived behavioral indicators.

Three gaps follow. First, the behavior-level structure linking phubbing to the components of love has not been described. Second, it is not known which elements act as central or bridging nodes between the two domains. Third, and most central, whether phubbing is associated uniformly or selectively with the perceived behaviors and the components of love, once their shared variance is taken into account, has not been established. The absence of a prior network model is not, in itself, the gap; total-score correlations cannot show whether phubbing relates uniformly or selectively to the components of love once their strong shared variance is partialled out, nor which specific perceived behaviors, rather than phubbing in general, account for any association. Addressing these questions is the theoretically meaningful information a network model can add beyond the established negative correlations. The present study addresses these gaps in a sample of adults in romantic relationships, a context in which phubbing has been studied at the individual level but not in relation to the components of love (Çizmeci, 2017; Karadağ et al., 2015). This context is theoretically relevant rather than incidental: cultural expectations about attentiveness during couple interaction, about marriage, and about acceptable public and private phone use may shape both how much phubbing is perceived and how strongly it is tied to relationship evaluations, so the associations reported here may be specific to this setting. Its aims were to identify the strongest conditional associations among the nine phubbing behaviors, the three components of love, and global relationship quality; to determine the most central nodes; to identify bridge nodes; and to examine the accuracy, stability, and robustness of the network. The contribution is twofold: substantively, the study locates phubbing within the structure of love rather than relating it to a single outcome; methodologically, it reports the stability of every estimate, so that firm structural conclusions can be separated from tentative behavior-level ones. On this basis, as exploratory expectations rather than confirmatory hypotheses, we anticipated that (H1) the phubbing behaviors and the love components would form two distinct but negatively connected communities; (H2) the cross-community associations of phubbing with intimacy and with global relationship quality would be larger in absolute value than its associations with passion and commitment; and (H3) the elements linking the two communities would reflect attention during shared interaction rather than the mere presence of the phone. We did not form a directional hypothesis about relationship status; possible differences between married and dating participants were examined only exploratorily. Because the design is cross-sectional, all associations are treated as correlational, and these expectations are examined descriptively rather than as confirmatory tests.

## 2. Methods

### 2.1. Study Design and Reporting Standard

This cross-sectional correlational study examined the network structure of partner phubbing and triangular love among adults in romantic relationships. The study was reported in accordance with the STROBE (Strengthening the Reporting of Observational Studies in Epidemiology) checklist for cross-sectional studies; the completed checklist is provided in the Supplementary Materials.

### 2.2. Setting and Participants

Data were collected using a structured online self-report questionnaire administered through Google Forms. The survey was distributed online and completed between May and July 2025. The survey link was circulated through the researchers’ personal contacts and networks. Participants were recruited by convenience sampling from adults living in various provinces of Turkey who were currently in a romantic relationship or married. Eligible participants were individuals who (a) were 18 years or older, (b) were currently in a romantic or marital relationship, and (c) provided informed consent. Participation was voluntary and anonymous, and no compensation was provided. Of the 476 participants who completed the survey, 472 had complete data on all network variables and formed the analytic sample for the network model. Because the survey was anonymous, the numbers of individuals who viewed the link, declined consent, or began but did not complete the survey could not be recorded; the figures reported therefore distinguish the 476 completers from the 472 participants retained in every network analysis. Duplicate submissions were limited by the survey’s one-response-per-account setting, which required respondents to sign in and allowed a single submission per Google account while collecting no identifying information. Couple membership was not assessed, so both members of some couples may have responded; the assumption of independent observations cannot be fully verified, and this is noted as a limitation.

### 2.3. Sample Size Consideration

Consistent with methodological recommendations for psychometric network analysis, no a priori power analysis was conducted, as power analysis is not well defined for regularized network models; instead, the sample is described as pragmatic, with the bootstrap results used as post hoc evidence about the stability of particular estimates (Epskamp et al., 2018). Precision and stability varied across the results rather than being uniformly high. Expected-influence centrality was stable (CS-coefficient = 0.75) and edge weights were estimated with moderate precision (mean 95% confidence-interval width = 0.14), whereas bridge centrality was only moderately stable (CS-coefficient = 0.45) and every individual cross-community edge interval included zero. The sample size is therefore treated as pragmatic rather than as evidence of general adequacy: counting observations per possible edge does not provide a valid power or precision guarantee for a regularized network, and the bootstrap results speak only to the stability of particular estimates, not to the reliability of the weak cross-community associations. A simulation-based analysis tied to a minimum edge of interest would be needed to justify a target sample size.

### 2.4. Measures

#### 2.4.1. Partner Phubbing Scale

Perceived partner phubbing was assessed using the Partner Phubbing Scale developed by Roberts and David (2016) and adapted into Turkish by Çizmeci (2017). The scale contains nine items rated on a 5-point scale, with higher scores indicating greater perceived phubbing. Because respondents rate a partner’s conduct, these items index perceived rather than directly observed behavior. The nine items served as behavior-level nodes; one reverse-keyed item was recoded so that higher scores indicated more phubbing throughout. In the present sample, Cronbach’s α was 0.80.

#### 2.4.2. Triangular Love Scale

Triangular love was assessed using the Triangular Love Scale developed by Sternberg (1997) and adapted into Turkish by Soyer and Gizir (2021). The scale measures intimacy, passion, and commitment on a 9-point scale. The three subscale scores served as nodes. Cronbach’s α values were 0.96 for intimacy, 0.94 for passion, and 0.96 for commitment.

#### 2.4.3. Perceived Romantic Relationship Quality Scale

Global relationship quality was assessed using the Perceived Romantic Relationship Quality Scale developed by Fletcher et al. (2000) and adapted into Turkish by Sağkal and Özdemir (2018). This six-item, 7-point measure (α = 0.89) provided a fourth relationship node. Its items index satisfaction, commitment, intimacy, trust, passion, and love, so the measure is treated here as a global evaluative summary of relationship quality rather than as an index of any single component. For all three instruments, items were rated on Likert-type scales (partner phubbing, 1–5; triangular love, 1–9; relationship quality, 1–7), and scale scores were formed as described above; for example, one phubbing item asks how often the partner checks the phone during a shared meal. The Turkish adaptations used here reported evidence of structural validity, including confirmatory factor analyses, in their validation studies (Çizmeci, 2017; Soyer & Gizir, 2021; Sağkal & Özdemir, 2018). Because we report internal consistency but did not re-estimate the factor structure in the present sample, the psychometric evidence provided here is partial; item-level descriptive statistics for the phubbing items are reported in the Supplementary Materials.

### 2.5. Bias Control

To minimize potential sources of bias, standardized and validated instruments were used to reduce measurement bias, and structured questionnaires ensured consistency in data collection. A reverse-keyed phubbing item was recorded before analysis. Because data were collected by self-report, recall bias and social desirability bias cannot be excluded. Statistical regularization was applied during network estimation to reduce spurious associations, and conditional-dependence modeling was used to estimate each association while controlling for all other variables in the network. These procedures do not address selection bias, common-method or single-informant bias, social-desirability bias, reverse causation, or omitted-variable confounding; regularization and conditional-dependence modeling reduce spurious associations but are not bias controls for these threats, which remain unresolved and are considered in the Discussion.

### 2.6. Missing Data

Four of the 476 participants (0.8%) had missing data on one or more network variables and were excluded, yielding an analytic sample of 472. Little’s test indicated that these values were consistent with a missing completely at random pattern, χ^2^(4) = 6.44, *p* = 0.169, so listwise exclusion is unlikely to have introduced systematic bias. Little’s test was computed on the composite study variables (partner phubbing, intimacy, passion, commitment, and relationship quality), which determines its degrees of freedom; only four cases had any missing values, so variable-specific missingness was minimal, and the small number of excluded cases precluded a meaningful comparison of their characteristics with the analytic sample.

### 2.7. Statistical Analysis

#### 2.7.1. Preliminary Analyses

Descriptive statistics were computed for demographic characteristics and for each study variable, and reliability was examined using Cronbach’s α. The distribution of all variables was inspected. Shapiro–Wilk tests were significant for every variable (*p*s < 0.001), but at n = 472 these tests detect even trivial departures, so skewness and kurtosis were examined as well: absolute skewness did not exceed 1.33 and absolute excess kurtosis did not exceed 1.23, indicating moderate rather than severe non-normality. Spearman correlations were therefore used for the primary network, and a polychoric–polyserial correlation matrix appropriate for the ordinal phubbing items was estimated as a sensitivity analysis. Three considerations led us to retain the Gaussian graphical model on Spearman correlations as the primary specification rather than the polychoric alternative. Polychoric estimation assumes a normally distributed latent variable behind each ordinal response, an assumption that cannot be verified with the present data. The love facets and global relationship quality are continuous composites, so a fully polychoric matrix is not defined for this set of nodes; a mixed polychoric–polyserial matrix must instead be assembled pairwise and then adjusted to positive definiteness, which introduces an approximation of its own. Most important, the two specifications yielded almost indistinguishable networks, so no substantive conclusion depends on the choice. The nine phubbing items were modeled as separate nodes rather than as a single summed score, because the aim was to identify which specific behaviors are most connected to romantic experience, and because the items describe behaviorally distinct acts (for example, using the phone during a meal, mid-conversation, or when out) rather than near-synonymous paraphrases. Because these items nevertheless belong to a single scale and show some local dependence, the resulting edges are treated as conditional associations among indicators and are not interpreted as evidence that individual behaviors carry independent psychological mechanisms. The theoretically defined love components and global quality were modeled at the subscale level. Modeling one construct at the item level and the others at the subscale level is a deliberate choice that we return to in the Limitations; regularization during estimation and the reporting of node predictability help to gauge whether local dependence among the phubbing items inflated within-community connectivity. Three considerations support this asymmetry. First, the research question concerns which enacted behaviors are connected to romantic experience, and that question cannot be posed once the behaviors are summed. Second, the components of Sternberg’s model are defined theoretically at the facet rather than the item level, so subscale scores are their natural representation. Third, adding the 24 love items would have produced a 37-node network with fewer than two observations per estimable parameter, well below the requirements for stable estimation at this sample size. To verify that the asymmetry did not drive the results, a fully subscale-level network in which phubbing was represented by its total score was also estimated. Node redundancy was additionally examined before estimation with a goldbricker-type procedure, in which two nodes are flagged as potentially redundant when fewer than 25% of their correlations with the remaining nodes differ significantly. This procedure follows the logic of unique variable analysis, which flags variables that are locally dependent and therefore only partly distinct (Christensen et al., 2023).

#### 2.7.2. Network Estimation

A psychometric network was modeled as a Gaussian Graphical Model consisting of 13 nodes: nine partner-phubbing behaviors and four relationship constructs (intimacy, passion, commitment, and global relationship quality). Edges represented regularized partial correlations between two nodes after conditioning on all other nodes. Formally, the precision matrix Θ (the inverse of the covariance matrix) was estimated by minimizing the ℓ1-penalized objective minΘ (tr(SΘ) − log det(Θ) + γ Σi ≠ j |θij|), where S is the sample covariance matrix (here the Spearman correlation matrix), the standardized off-diagonal elements of Θ are the partial correlations represented by the edges, and γ is the EBIC tuning parameter (set to 0.5). Because the variables departed from multivariate normality, rank-based (Spearman) correlations were used to construct S rather than Pearson covariances; this places the model in the nonparanormal, or semiparametric Gaussian copula, family, which relaxes the normality assumption by using rank correlations to recover the latent Gaussian dependence structure rather than treating the ordinal responses as exactly Gaussian (Liu et al., 2012). The network was estimated using the graphical least absolute shrinkage and selection operator (graphical LASSO) with model selection based on the Extended Bayesian Information Criterion (EBIC), with the tuning parameter (γ) set to 0.5, the value recommended for favoring sparse recovery in psychological networks (Foygel & Drton, 2010); sensitivity to this choice was examined by re-estimating the network with γ = 0 and γ = 0.25. In the network visualization, green edges indicate positive associations and red edges indicate negative associations, and thicker edges represent stronger regularized partial correlations. The 13 nodes were assigned a priori to two communities, partner phubbing and relationship, because the two sets index conceptually different phenomena (enacted behaviors versus relational constructs). The two node sets also differ in level of aggregation, with nine item-level phubbing nodes versus four composite relationship nodes; this asymmetry can itself produce dense within-set connections and a two-community split, so the community structure is partly measurement-structured. The mixed-level network is therefore treated as exploratory, and a balanced, fully subscale-level network is reported as a sensitivity analysis. To corroborate this structure, data-driven community detection was also conducted. Greedy modularity optimization served as the primary algorithm because it is deterministic and requires no resolution parameter (Clauset et al., 2004). Because community solutions can depend on the algorithm chosen, the Louvain and label-propagation algorithms were run for comparison.

#### 2.7.3. Centrality Indices and Bridge Centrality

To evaluate the relative importance of each node, strength and expected influence were calculated. Strength was defined as the sum of the absolute edge weights connected to a node, whereas expected influence was the sum of signed edge weights, making it suitable for networks containing both positive and negative edges (Robinaugh et al., 2016). Bridge strength and one-step bridge expected influence were calculated to identify nodes connecting the two communities (Jones et al., 2021). Bridge strength quantified the absolute magnitude of a node’s cross-community connections, whereas bridge expected influence retained their sign. Centrality and bridge centrality values were standardized as z-scores for comparison. Node predictability, the proportion of each node’s variance explained by its neighbors, was also computed as an absolute measure of interconnectedness (Haslbeck & Waldorp, 2018). Regularization and node predictability do not remove local dependence among items of the same scale; approaches such as residual or latent network modeling, or prior dimension reduction, would be needed to resolve it, so centrality and community results should be read with this caveat.

#### 2.7.4. Network Accuracy and Stability

The accuracy of edge weights was assessed using nonparametric bootstrap procedures with 1000 bootstrap samples, generating 95% confidence intervals for edge weights. The stability of centrality indices was evaluated using a case-dropping bootstrap procedure, quantified with the correlation-stability (CS) coefficient; a CS-coefficient above 0.25 is considered acceptable and above 0.50 indicates good stability (Epskamp et al., 2018). Bootstrapped difference tests were also computed to examine whether individual edge weights and centrality indices differed reliably from one another. For computational feasibility, bootstraps used the regularization parameter selected in the full sample.

#### 2.7.5. Sensitivity Analysis

Six sensitivity analyses were conducted, each retaining the primary estimation procedure. First, the network was re-estimated from Pearson correlations. Second, it was re-estimated from a polychoric–polyserial correlation matrix appropriate for ordinal items. Third, the EBIC tuning parameter was varied between 0 and 0.5. Fourth, a fully subscale-level network was estimated in which phubbing was represented by its total score. Fifth, because relationship status was heterogeneous, the networks of married and dating participants were compared with a permutation-based network comparison test (500 permutations) of structure invariance and global strength invariance. This subgroup comparison was not prespecified or powered for network inference; the permutation count and moderate subsample sizes limit its precision, and it is treated as exploratory. Sixth, because the redundancy analysis indicated possible overlap between intimacy and global relationship quality, the network was re-estimated after removing global relationship quality, leaving 12 nodes. Each alternative network was compared numerically with the primary network in terms of edge weights. For the ordinal-item sensitivity network, polychoric and polyserial correlations were computed pairwise, assembled into a single matrix, and adjusted to the nearest positive-definite matrix by clipping negative eigenvalues before estimation; because the primary model used five-category ordinal items as nodes in a Gaussian graphical model, this network serves as a check on the consequences of that choice. Because participant consent did not cover public posting of the data, the de-identified data are not deposited in an open repository; they are available from the corresponding author on reasonable request, subject to the constraints of the ethics approval. The analysis code, which records the exact functions, package versions, and random seeds used for the bootstraps, is available from the corresponding author on request.

### 2.8. Ethical Considerations

Ethical approval was obtained from the Mersin University Scientific Research and Publication Ethics Committee (approval date: 20 May 2025, approval No. 138). All participants provided informed consent prior to participation. All procedures were conducted in accordance with the Declaration of Helsinki and its later amendments.

## 3. Results

### 3.1. Participant Characteristics

A total of 476 adults in romantic relationships were included. These 476 were the survey completers; all network analyses were conducted on the 472 participants with complete data on every network variable. The mean age was 33.1 years (SD = 8.5, range 19–58), and 82.6% were women. Regarding marital status, 55.7% were married and 44.3% were in a dating relationship. Relationship duration was assessed, but 290 participants (61%) did not provide it; among the 186 who did, the median was 10 years (interquartile range 4 to 17). Given this level of missingness, imputing or modelling relationship duration could have biased the estimates, so it was not entered into the network. Its role is nonetheless likely to be substantive, because the meaning of phubbing, intimacy, and commitment may differ between early-stage and long-term relationships; we therefore treat relationship duration as a priority moderator for future studies designed to collect it completely. Other characteristics relevant to phone use and relationship functioning, such as education, socioeconomic status, province, cohabitation, and children, were not recorded, which limits assessment of representativeness and potential confounding. Detailed sociodemographic characteristics are shown in Table 1.

Descriptive statistics and reliabilities of the study variables are presented in Table 2. At the zero-order level, higher phubbing was associated with lower scores on every relationship construct (Spearman ρs = −0.25 to −0.33, all *p* < 0.001), and the relationship constructs were strongly intercorrelated among themselves (ρs = 0.83–0.90). Absolute skewness reached 1.33 and absolute excess kurtosis 1.23. Because the correlations among the relationship constructs were high, node redundancy was examined before estimation. Five of the six pairs of relationship nodes showed distinguishable patterns of association with the remaining nodes (36% to 82% of their correlations differed significantly); only the intimacy–quality pair fell below the 25% criterion (18%), indicating possible redundancy. Several phubbing item pairs also fell below this criterion, consistent with the local dependence expected among items of a single scale.

### 3.2. Network Structure

**Figure 1:**
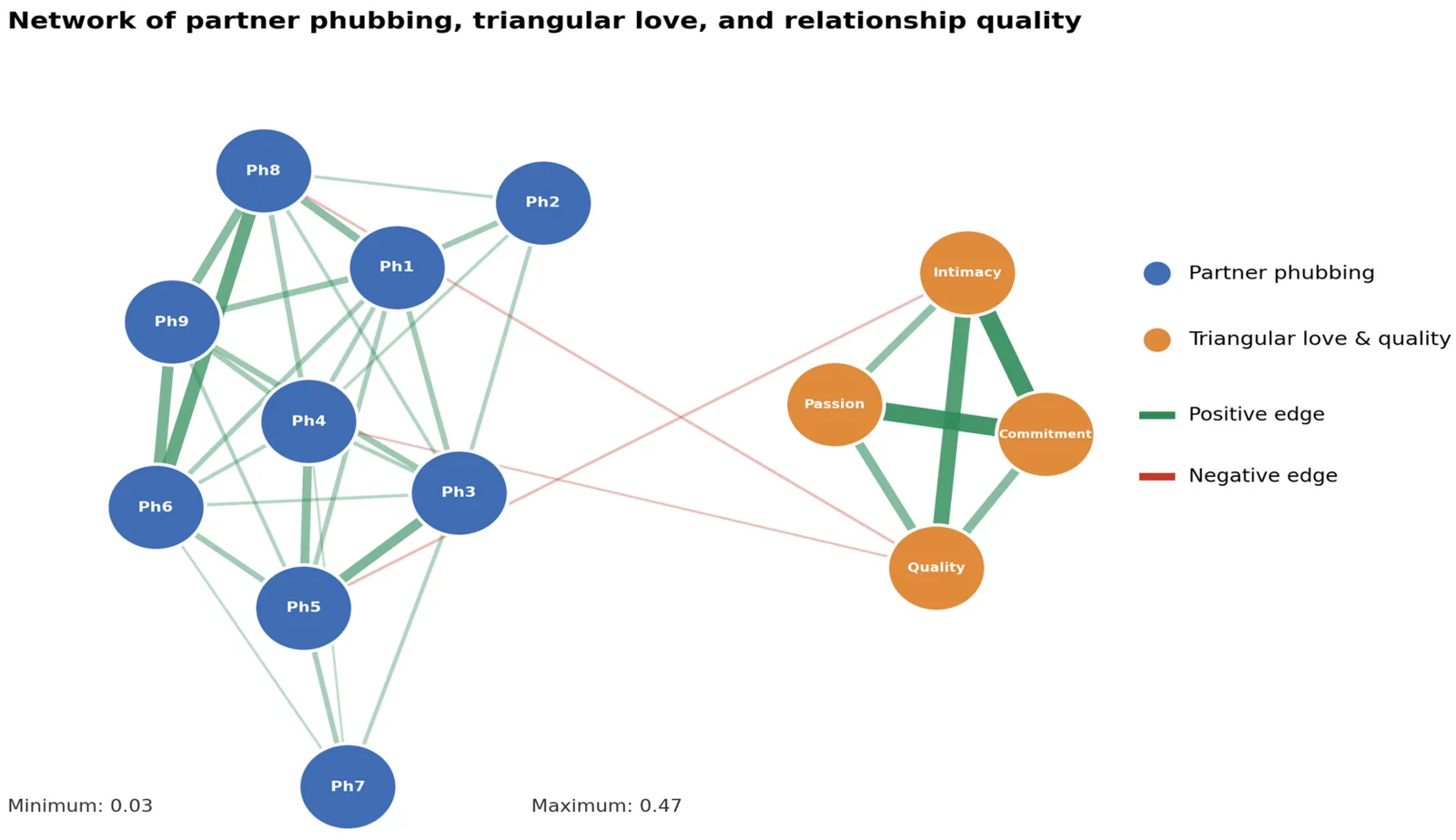
Network structure of partner phubbing and triangular love.

The estimated network contained 50 nonzero edges among the 13 nodes (edge density = 0.64; Figure 1). The nine phubbing behaviors and the four relationship constructs formed two internally dense, coherent communities, with strong positive connections within each community and only a small number of weak, exclusively negative edges between them, so that more phubbing co-occurred with lower love and quality. Although EBICglasso favors sparsity, the retained network was comparatively dense (50 of 78 possible edges); this density is carried almost entirely by the strong positive associations within each community and by the measurement overlap among the relationship constructs, rather than by many phubbing–love links, of which only a few small negative edges were retained. The strongest cross-community associations were between glancing at the phone while talking and intimacy (partial r = −0.05), using the phone when out together and global quality (−0.05), and checking the phone mid-conversation and both quality and passion (−0.04 and −0.03). Data-driven community detection was consistent with the two theoretical communities (modularity = 0.41), supporting rather than merely assuming the two-group structure. Because the two communities also correspond to the two measurement instruments and to the two levels of aggregation, separation by measurement source cannot by itself establish that phubbing and love form distinct substantive systems. Greedy modularity optimization and the Louvain algorithm both recovered exactly these two communities, whereas label propagation returned a single community, an outcome typical of that algorithm in dense weighted graphs. Because the three algorithms did not fully agree, the two-community solution is best read as supported by two of three procedures rather than as unequivocal. Bootstrapped difference tests indicated that 47% of the pairs of non-zero edges differed reliably from one another, so the ordering of individual edges is only partly determinate.

Nodes represent the measured behaviors and constructs, and node colours indicate communities: partner phubbing (blue) and triangular love/quality (orange). Green edges indicate positive regularized partial correlations and red edges indicate negative regularized partial correlations; thicker edges represent stronger associations. Because edge width is proportional to the absolute partial correlation, the strong within-community associations dominate the figure visually; the cross-community (phubbing–love) edges are very small (|partial r| ≤ 0.05) and should not be read as indicating strong associations. “Minimum” and “Maximum” denote the edge-weight values mapped to the thinnest and thickest edges. Abbreviations: Ph1, uses phone during a shared meal; Ph2, keeps phone visible when together; Ph3, holds phone when I am beside them; Ph4, checks phone mid-conversation; Ph5, glances at phone while talking; Ph6, uses phone during shared free time; Ph7, uses phone while talking (reverse-recoded); Ph8, uses phone when out together; and Ph9, uses phone when the conversation lulls.

### 3.3. Centrality Analysis

**Figure 2:**
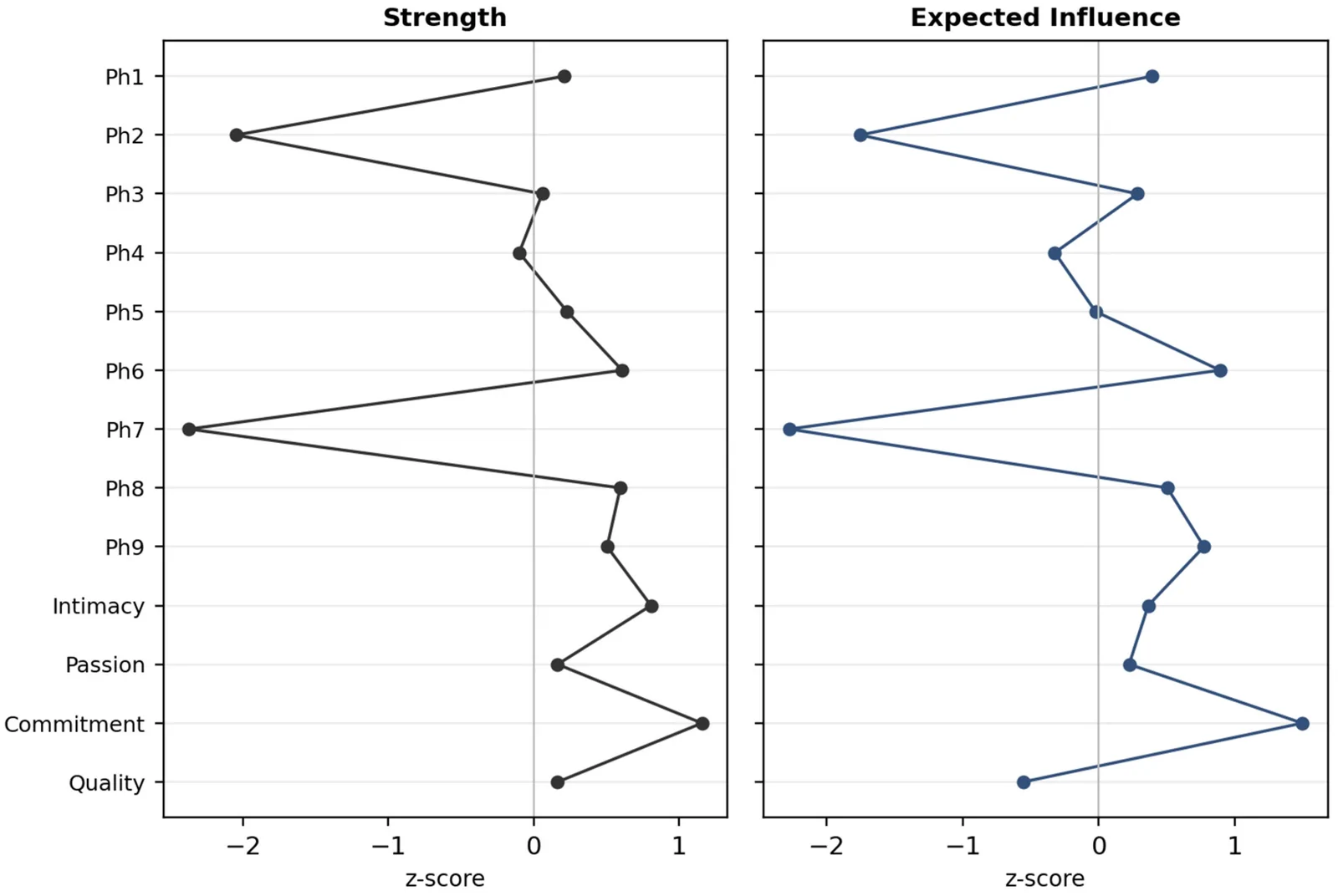
Standardized strength and expected-influence centrality of the 13 nodes. Higher values indicate greater relative connectivity within the estimated network.

Centrality indices are presented in Figure 2, and numerical centrality and bridge values are reported in Supplementary Table S2. Expected-influence centrality was highest for commitment and for several core phubbing behaviors (using the phone during shared free time, when the conversation lulls, and when out together), reflecting their dense within-community ties. On average, neighboring nodes explained 79% of the variance in each relationship construct and 37% in each phubbing behavior (node predictability). Predictability of this magnitude is unusual and most plausibly reflects the conceptual overlap among the love facets and global relationship quality rather than an unusually well-determined system; as reported above, however, this overlap did not amount to statistical redundancy for most pairs. Centrality reflects statistical connectivity within the estimated network and should not be interpreted as causal or definitive practical importance.

### 3.4. Bridge Centrality

**Figure 3:**
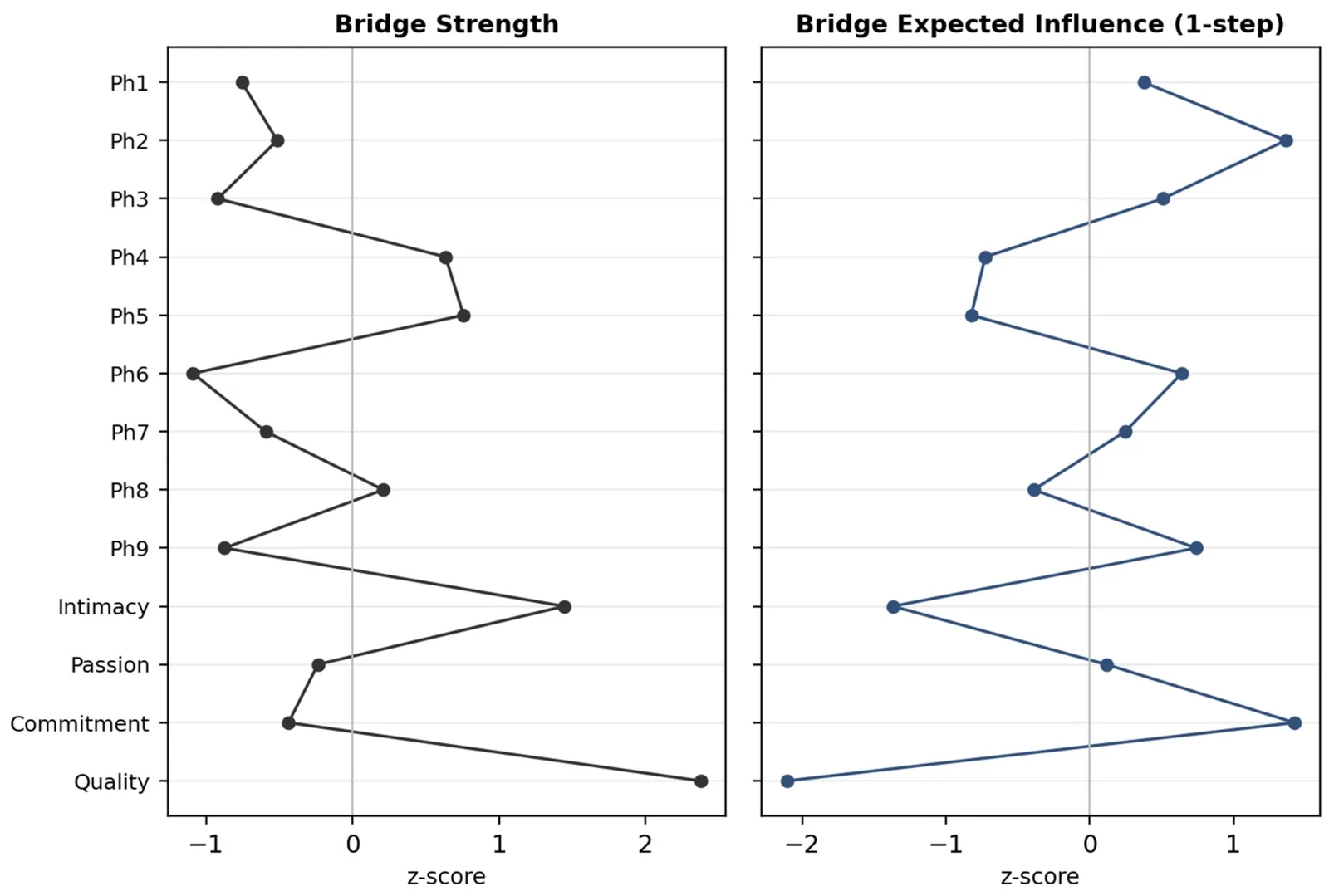
Standardized bridge strength and one-step bridge expected influence of the 13 nodes across the two communities. Higher values indicate greater cross-community connectivity.

Bridge centrality indices are presented in Figure 3. On the relationship side, global quality and intimacy were the constructs most connected to the phubbing community; passion and commitment were more peripheral to it. On the behavior side, the behaviors that reflect phone use during interaction (glancing at the phone while talking, checking it mid-conversation, and using it when out together) showed relatively higher estimated cross-community (bridge) associations, whereas merely keeping the phone visible or in hand showed lower ones. In absolute terms these cross-community estimates were very small (about −0.03 to −0.05); their magnitude and uncertainty should be kept in view before any behavior is called a bridge. These behavior-level differences should be interpreted cautiously: bootstrapped difference tests indicated that expected-influence centrality was moderately differentiated (51% of node pairs differed significantly), whereas bridge expected influence was weakly differentiated (only 27% of pairs), so only broad distinctions rather than fine rankings among behaviors are statistically supported. Standardized centrality and bridge values are reported in Supplementary Table S2, and the raw indices scale monotonically with them. Bridge strength sums the absolute cross-community edges, whereas bridge expected influence sums their signed values, so a node’s standardized bridge expected influence can be positive even when its cross-community edges are predominantly negative; the two indices should not be read interchangeably.

### 3.5. Network Accuracy and Stability

Nonparametric bootstrap analysis showed that the 50 nonzero edges were estimated with moderate precision (mean 95% confidence-interval width = 0.14; Supplementary Figure S1). No individual cross-community (phubbing–relationship) edge had a bootstrap confidence interval that excluded zero, so, edge by edge, the cross-community links are weak and not distinguishable from zero; because no omnibus test of an aggregate bridge was performed, they are described only as a set of uniformly negative edges rather than as a reliable aggregate pattern. Case-dropping analysis indicated that expected-influence centrality was highly stable (CS-coefficient = 0.75), whereas bridge expected influence was less stable (CS-coefficient = 0.45), exceeding the 0.25 threshold for acceptable stability but falling below 0.50 (Supplementary Figure S2). Accordingly, the ordering of the most central nodes is resampling-stable within this sample, whereas claims about which particular behaviors show the highest estimated cross-community associations remain exploratory.

### 3.6. Sensitivity Analysis

The Pearson-based sensitivity network was numerically almost identical to the primary Spearman-based network (edge-weight r = 0.98), with the same community structure and the same relationship-side nodes (quality and intimacy) nearest the phubbing community. The polychoric–polyserial network, which treats the phubbing items as ordinal, was likewise almost identical (edge-weight r = 0.98). These edge-weight correlations are dominated by the numerous within-community edges; the much smaller set of cross-community edges is less consistent across correlation methods and, as reported above, individually includes zero, so the high overall similarity should not be read as stability of the phubbing–love links specifically. Varying the EBIC tuning parameter between 0 and 0.5 left the selected penalty and the resulting edge set unchanged (edge-weight r = 1.00). In the fully subscale-level network, phubbing again showed negative edges with global quality (partial r = −0.11) and intimacy (−0.05) and no edge with passion or commitment, reproducing the pattern obtained at the item level. Finally, the network comparison test indicated that overall connectivity did not differ between married and dating participants (global strength difference = 0.011, *p* = 0.944), whereas the test of structure invariance was significant (largest edge difference = 0.29, *p* = 0.016), suggesting that individual edges may differ by relationship status. The global-strength and structure-invariance test statistics are reported above; because the comparison was exploratory and uncorrected for the number of edges, individual edge differences are not interpreted here. This subgroup comparison was exploratory, the subsamples were of moderate size, and the edge-level differences were not corrected for multiplicity. Removing global relationship quality left the structure essentially unchanged: the resulting 12-node network contained 45 edges whose weights correlated r = 0.99 with the corresponding edges of the full network, and the ordering of the phubbing behaviors by bridge expected influence was almost identical (Spearman ρ = 0.98). Intimacy absorbed the connective role it had previously shared with global quality, its bridge expected influence increasing from −0.09 to −0.14, whereas commitment remained near zero. The findings therefore do not depend on retaining both constructs. Taken together, the broad, construct-level pattern did not depend on the correlation method, the tuning parameter, or the level at which phubbing was modeled, although it may not be fully invariant across relationship status. Because the subscale-level network represents phubbing by a single total score, it cannot evaluate the behavior-level bridge findings, which rest only on the item-level network and are not validated by that analysis.

## 4. Discussion

This study modeled partner phubbing and triangular love as one connected system, and three findings stand out. First, the phubbing behaviors and the love components formed two partly measurement-structured groups, supported by two of the three community-detection algorithms; the same division was recovered when the analysis was repeated with a different correlation method. Because the two groups align with the two measurement instruments and with the two levels of aggregation used to build the network, this division is best read as a partly measurement-structured pattern that requires replication with balanced node definitions before it is treated as a substantive discovery. Second, relationship quality and intimacy were the components most closely connected to phubbing, whereas passion and commitment were more distant. These two received the largest point estimates within an uncertain set; because no individual cross-community edge excluded zero, this is a difference in point estimates rather than an established difference in connection. Because intimacy and global quality overlapped substantially, this finding is better read as phubbing being most connected to a single closeness-and-evaluation dimension than to two independent facets. Third, at the level of individual behaviors, the acts that reached across to romantic experience were those that interrupt attention during shared time, although these specific connections were weak and could not be established with confidence. Taken together, the results describe phubbing in more differentiated terms than a single summary score allows, while reporting how far each part of the description can be trusted.

Both groups were internally cohesive. Within the relationship group, intimacy, commitment, and overall quality moved closely together and were highly predictable from one another, so that for these participants the facets of love appear to function less as separate dimensions than as one integrated appraisal; this is consistent with the high intercorrelations commonly reported among relationship quality components (Fletcher et al., 2000). The redundancy analysis sharpened this observation by flagging one pair, intimacy and global relationship quality, as possibly redundant, which raises the question whether the two are separate constructs at all. Conceptually they are distinguishable. Intimacy is one component of love in Sternberg’s model and refers to felt closeness, warmth, and disclosure, whereas relationship quality is an evaluative summary that also draws on satisfaction, trust, and commitment (Fletcher et al., 2000; Sternberg, 1986). Empirically, only this pair fell below the redundancy criterion, and removing global quality changed neither the community structure nor the ordering of the behaviors, with intimacy simply absorbing the connective role the two had shared. The most defensible reading is therefore not that the constructs are identical, but that respondents completing a single self-report battery do not draw a sharp line between feeling close to a partner and judging the relationship to be going well, so that a common evaluative disposition plausibly underlies both. Statements about intimacy in this network are best read as statements about closeness-related appraisal rather than about a facet cleanly separated from global satisfaction. Whether all four relationship constructs should be modeled together is, then, a fair question. We retained them because they are conceptually distinct in their source theories and because the sensitivity network without global quality reproduced the same structure; the high overlap nonetheless means that intimacy and global quality are better understood as closely related indicators of a common evaluative dimension than as fully independent nodes (Christensen et al., 2023), and any result involving either should be read with that overlap in mind. More broadly, correlations as high as 0.90 among intimacy, passion, commitment, and global quality raise a question of discriminant validity and suggest that a general positive relationship-evaluation factor, rather than four distinct components, may underlie the relationship nodes; such a factor would also account for their unusually high predictability. Within the phubbing group, behaviors involving active phone use during shared time were the most interconnected, while keeping the phone visible or in hand was more peripheral. Connections between the two groups were few and consistently negative, which agrees with earlier reports (Beukeboom & Pollmann, 2021; McDaniel & Coyne, 2016; Ni et al., 2025; Yam, 2023).

Those cross-group connections were small, but this is a feature of the model rather than evidence that phubbing is unimportant. Network edges are partial correlations that hold all other variables constant, so the strong associations within each group absorb much of the shared variance and leave a modest remainder between groups. These weak conditional associations are, however, themselves an empirical result: it remains possible that, once the shared variance among the relationship constructs is removed, phubbing has little unique association with any single component, and we present the small conditional edges as a finding to be explained rather than only as an artifact of the method. At the zero-order level, phubbing correlated with the relationship measures between about −0.25 and −0.33, close to the pooled estimates reported in meta-analytic work on partner phubbing (Ni et al., 2025), which suggests that the present sample is not unusual in this respect. The contribution of the network lies in the conditional dependence structure it reveals rather than in the magnitude, or the presumed importance, of any single edge. A regularized network shows which associations remain once every other variable is held constant; it does not show which associations are strongest or most meaningful in an absolute sense. The attenuation of the cross-domain associations after conditioning should therefore be read partly as a consequence of the strong multicollinearity within the relationship domain, which absorbs shared variance, rather than as evidence that phubbing is only weakly related to romantic experience.

The ordering of the love components is consistent with current accounts of phubbing. Intimacy and overall quality rest on feeling attended to and valued and were closely connected to phubbing, whereas passion and commitment draw on desire and on longer-term decisions and were more peripheral. Phubbing has been described as a repeated violation of the attention a partner expects during shared moments (Kelly et al., 2017; Roberts & David, 2016), and experimental work shows that being phubbed lowers perceived communication quality and threatens social needs (Chotpitayasunondh & Douglas, 2018). Such moments may reflect, rather than establish, how close and how satisfied a partner feels, and they appear to bear less on attraction or on the decision to remain. The high predictability of the relationship group is consistent with this reading, but it should not be taken to mean that the relational system is determined by neighboring nodes, since predictability of this magnitude largely reflects the conceptual overlap among the relationship constructs.

On the behavior side, the indicators with relatively higher estimated cross-community associations were those reflecting attention during interaction rather than the mere presence of the device. Read as a post hoc interpretation, this is consistent with the view that the cost of phubbing lies in enacted inattention rather than in the phone itself (Chotpitayasunondh & Douglas, 2018; Roberts & David, 2016). It should not be taken as evidence that particular behaviors are distinct targets, because the cross-community associations were very weak, did not individually exclude zero, and rested on only moderately stable bridge estimates. Any behavioral reading is therefore hypothesis-generating rather than established. Such readings are exploratory and statistical rather than evidence of a causal or mechanistic process. Consistent with this, only 27% of the pairwise comparisons of bridge expected influence differed reliably, so the apparent primacy of interaction-disrupting behaviors is best stated as an unconfirmed hypothesis.

The present results align with the variable-centered literature in direction while adding resolution. Studies based on total scores report negative associations between partner phubbing and relationship satisfaction or quality, with modest effect sizes (Ni et al., 2025; Roberts & David, 2016; Yam, 2023), and the zero-order correlations reported here fall in the same range; what the network adds is a division of that association across behaviors and facets. This added resolution should be regarded as provisional: because the item-level cross-community associations were weak and not individually reliable, the advantage over total-score approaches is demonstrated only for the stable structural features, not for the specific behavioral bridges, which would need to be shown stable, distinguishable, and replicable before such an advantage is claimed. Earlier work has shown that technoference relates to conflict over device use, which in turn relates to satisfaction (McDaniel & Coyne, 2016), and that relationship satisfaction carries part of the association between partner phubbing and well-being (Wang et al., 2017). Both findings place relational appraisal in an intermediate position, consistent with the central place of relationship quality and intimacy in the present structure.

### 4.1. Implications

Read conservatively, the findings carry a modest message. Intimacy and the overall appraisal of the relationship had the largest cross-community point estimates, but these fell within an uncertain set in which no individual edge could be distinguished from zero; any support for couples that this might suggest, such as protecting closeness and attentive shared time rather than targeting phone use in general, is therefore not established by the present estimates. It would be premature to recommend specific behavioral targets; given the weakness of the cross-community associations noted above, candidates such as device-free conversations and outings are best regarded as hypotheses for intervention research. Experimental or daily diary studies that manipulate or track phone use during shared time would be needed before any such recommendation could be supported. It may also help partners to read phubbing as a signal about attention rather than only as a habit, since interpretation may shape how the behavior is experienced (Beukeboom & Pollmann, 2021; Kelly et al., 2017); and because phubbing tends to become normalized within couples (Chotpitayasunondh & Douglas, 2016), agreements made early may be easier to sustain than corrections made later. Any such agreements should also leave room for reasonable accommodation. Because some device use reflects information-processing needs rather than disengagement, for example, using a phone to manage sensory load or to sustain attention, a blanket norm that equates putting the phone away with love risks excluding partners who rely on devices to communicate or self-regulate. Agreements that permit legitimate, need-based phone use during interaction are therefore preferable to uniform prohibitions. The practical points above are therefore offered as directions for future intervention research, not as recommendations for current practice. More broadly, if the equation of phone use with a lack of affection becomes entrenched, whether within couples or in wider social norms, people who depend on devices for information processing risk being structurally excluded from forming and sustaining intimate relationships. Reasonable accommodation therefore means more than granting permission. It involves partners aligning their interpretations, recognizing that glancing at a device during a conversation does not necessarily mean one is not listening, and agreeing on explicit communication rules, such as saying that one needs to check a phone for five minutes to reduce sensory overload, so that need-based use is understood rather than read as rejection.

The study also illustrates what a behavior-level network adds. A single phubbing score would show only a weak negative association with a global outcome, whereas the network located that association among particular behaviors and facets and separated stable elements of the structure from unstable ones. This carries a measurement implication: if phubbing behaviors differ in how strongly they relate to romantic experience, summing them may obscure as much as it summarizes, and instruments that distinguish attention-breaking items from indicators of mere device presence could prove more sensitive in relational research. The suggestion follows from the observed structure rather than from a formal measurement study and would require psychometric testing.

The comparison between married and dating participants should be read as exploratory. Overall connectivity did not differ between the groups, but the test of structure invariance was significant, which indicates that at least some network features differ by relationship status without, in itself, identifying which. The pattern was consistent with the differences lying within the relationship community rather than in its connection to phubbing, but this observation is tentative: the study was not designed or powered for subgroup-specific network inference, the subsamples were of moderate size, and the edge-level comparisons were not corrected for multiplicity. Detailed interpretation of individual edge differences is therefore not warranted. Relationship status is also confounded with relationship duration, cohabitation, age, and other unmeasured characteristics, so any difference cannot be attributed to marital status itself. Consistent with reports that relationship characteristics can condition phubbing associations (Wang et al., 2017), relationship status is best treated as a candidate moderator, to be examined in studies designed to estimate the two networks separately.

Further checks support the more stable conclusions. Data-driven community detection reproduced the two conceptually defined groups; a Pearson-based network was almost identical to the primary one (edge-weight r = 0.98); and expected-influence centrality was highly stable under case-dropping (CS-coefficient = 0.75). Bridge centrality was only moderately stable (CS-coefficient = 0.45) and no single cross-group edge could be distinguished from zero, so the structural findings are reported with greater confidence than the specific behavioral bridges.

### 4.2. Limitations

Several limitations should be kept in mind, and several of them bear on the estimated structure itself rather than only on its interpretation. In particular, local dependence among phubbing items, the mixed level of node aggregation, the redundancy between intimacy and global quality, single-informant measurement, and the disagreement among community-detection algorithms could each alter the recovered structure, and the findings should be weighed accordingly. The design is cross-sectional and cannot establish direction or timing; cross-lagged evidence suggests that device use can precede declines in satisfaction (Halpern & Katz, 2017), but longitudinal or daily diary networks would be needed to test this within the present structure. The cross-community connections were weak and bridge centrality only moderately stable, so the ordering of the behaviors by estimated cross-community association remains provisional and should be re-examined in new samples. All measures came from a single partner by self-report, which leaves room for common-method and single-informant bias; data from both members of a couple would allow stronger inferences. This is more than a routine caveat: because the same respondent rated both the partner’s phone use and the quality of the relationship, a general negative evaluative tendency could by itself produce the observed cross-domain associations, a central alternative to the attention-based interpretation. Phubbing was modeled at the item level and the love components at the subscale level, a deliberate asymmetry that future work could resolve by modeling both sides at a common level. Potential moderators, such as relationship length (Wang et al., 2017), were not examined, and the network was not compared across gender because few men participated. Redundancy analysis further suggested that intimacy and global relationship quality may be partly redundant and that several phubbing items showed local dependence, both of which inflate within-community connectivity and predictability. Although overall connectivity did not differ between married and dating participants, the structure-invariance test was significant, so the network may not be fully invariant across relationship status; this exploratory result requires replication. Finally, women were over-represented in this non-probability convenience sample from a single national context, which therefore cannot be regarded as representative of adults in Turkey. More specifically, because 82.6% of the sample were women, the findings most likely reflect women’s perceptions of a partner’s phubbing; given documented gender differences in how partners perceive their relationships, they may not extend to men or be symmetric within couples. Because recruitment relied on convenience sampling in a single country, it is also unclear whether patterns tied to smartphone-use norms generalize to other national or cultural contexts. Expectations about responsiveness during interaction, the acceptability of public phone use, and norms around marriage and gender roles may shape both how much phubbing is reported and what it is taken to mean, and these may differ across cultural settings.

## 5. Conclusions

Modeled as a network, partner phubbing and triangular love formed two partly measurement-structured groups, joined by a weak, negative link and recovered by two of the three community-detection algorithms. Overall relationship quality and intimacy, which overlapped and plausibly index a common evaluative dimension, were the facets most closely connected to phubbing. In this sample, however, no specific behavior-level cross-community connection was established with confidence. The elements with the highest estimated cross-community associations reflected attention during shared time rather than the presence of the phone, but these associations were too weak to identify specific behavioral targets. Compared with total-score approaches, this behavior-level view gives a more differentiated account of how phubbing relates to the components of love, although the added behavior-level differentiation was not statistically reliable. The findings describe statistical connectivity rather than causal influence. The specific behavior-level bridges remain tentative and hypothesis-generating. Both would benefit from confirmation in longitudinal and dyadic research.

## Figures and Tables

**Table 1:** Sociodemographic Characteristics of the Sample (N = 476).

Variable	Level	n (%) or Mean ± SD
Age, years		33.1 ± 8.5
Age group	19–24	73 (15.4)
	25–34	220 (46.3)
	35–44	124 (26.1)
	≥45	58 (12.2)
Gender	Women	393 (82.6)
	Men	83 (17.4)
Marital status	Married	265 (55.7)
	Dating	211 (44.3)

Notes: Categorical variables are presented as n (%) and continuous variables as mean ± SD. Age range: 19–58 years. One participant reported an implausible age and was excluded from the age statistics, so the four age categories sum to 475 rather than 476. Relationship duration was reported by 186 participants and is summarized in the text rather than tabulated.

**Table 2:** Descriptive Statistics and Reliability of Study Variables.

Scale	Items	Mean ± SD	Min–Max	Cronbach’s α
Partner phubbing (sum, 9–45)	9	25.0 ± 7.3	9–43	0.80
Relationship quality (sum, 6–42)	6	33.6 ± 7.4	6–42	0.89
Intimacy (item mean, 1–9)	9	7.4 ± 1.9	1–9	0.96
Passion (item mean, 1–9)	7	6.4 ± 2.1	1–9	0.94
Commitment (item mean, 1–9)	8	7.1 ± 2.0	1–9	0.96

Notes: Partner phubbing and relationship quality are total (sum) scores; intimacy, passion, and commitment are item means. Zero-order Spearman correlations among the composites are reported in Supplementary Table S1.

## Data Availability

The data and analysis code that support the findings of this study are available from the corresponding author upon reasonable request.

## Supplementary Materials

The following supporting information can be downloaded at https://www.mdpi.com/article/10.3390/bs16101810/s1, Supplementary File S1: STROBE Statement—Checklist for Cross-Sectional Studies STROBE. Table S1: Zero-order Spearman correlations among the study composites. Table S2: Node descriptions and standardized centrality and bridge centrality. Table S3: Item-level descriptive statistics for the nine partner-phubbing items. Figure S1: Bootstrapped 95% confidence intervals for all edge weights, ordered by the sample estimate. Cross-community (bridge) edges are highlighted in red and cluster near zero; every bridge interval includes zero. Figure S2: Case-dropping bootstrap stability of expected-influence and bridge expected-influence centrality. Expected influence remained highly stable; bridge centrality declined earlier, indicating lower stability.
