# Supplementary material for: Mapping Perceived Partner Phubbing, Triangular Love, and Relationship Quality: A Psychometric Network Analysis
Source: Behav Sci (Basel). 2026 Oct 3;16(10):1810. doi: 10.3390/bs16101810 (PMC13701943; doi:10.3390/bs16101810)
Supplement: Supplementary file 1 [file behavsci-16-01810-s001.zip › behavsci-4551913-supplementary.pdf]

Supplementary Material

Supplementary File S1. STROBE Statement—Checklist for Cross-Sectional Studies

Manuscript: Mapping Partner Phubbing, Triangular Love, and Relationship Quality: A Psychometric Network Analysis

Item descriptions below are abbreviated. The official STROBE checklist and its full item wording are available at https://www.strobe-statement.org. Page numbers refer to this revised manuscript; please verify them after final typesetting, since pagination can shift between word processors.

Item	Recommendation (abbreviated)	Reported in	Page
**Title and abstract**			
1a	State the study design in the title or the abstract	Abstract, Methods ("A cross-sectional study…")	1
1b	Give a balanced, informative summary of what was done and found	Structured abstract	1
**Introduction**			
2	Explain the scientific background and rationale	Introduction, opening paragraphs (prevalence, relational outcomes, attention-based mechanism, componential model of love)	2–5
3	State specific objectives, including any prespecified hypotheses	Introduction, final paragraph (four stated aims)	5
**Methods**			
4	Present key elements of the study design early in the paper	Methods — Study Design and Reporting Standard	6
5	Describe the setting, locations, and relevant dates of recruitment and data collection	Methods — Setting and Participants (online survey via Google Forms; provinces of Turkey; data collected May–July 2025)	6
6a	Give eligibility criteria and the sources and methods of participant selection	Methods — Setting and Participants (criteria a–c; convenience sampling)	6
7	Clearly define all outcomes, exposures, predictors, and potential confounders	Methods — Measures; Preliminary Analyses (13 network nodes: 9 phubbing behaviors, 3 love components, global relationship quality)	8–10
8	For each variable, give sources of data and details of measurement methods	Methods — Measures (three validated scales, reliabilities reported)	8–9
9	Describe any efforts to address potential sources of bias	Methods — Bias Control	9
10	Explain how the study size was arrived at	Methods — Sample Size Consideration	7

11	Explain how quantitative variables were handled in the analyses	Methods — Preliminary Analyses (item-level vs. subscale-level modelling and its justification; reverse-keyed item recoded; skewness and kurtosis inspected; goldbricker-type redundancy check)	10
12a	Describe all statistical methods, including those used to control for confounding	Methods — Network Estimation (EBICglasso, γ = 0.5, Spearman correlations; regularised partial correlations adjust for all other nodes); Centrality Indices and Bridge Centrality	12–13
12b	Describe any methods used to examine subgroups and interactions	Methods — Sensitivity Analysis (permutation-based network comparison test, 500 permutations: structure invariance and global strength invariance, married vs. dating)	14
12c	Explain how missing data were addressed	Methods — Missing Data (0.8% excluded; Little's MCAR test)	10
12d	If applicable, describe analytical methods taking account of the sampling strategy	Methods — Setting and Participants (non-probability convenience sample; no weighting applied)	6
12e	Describe any sensitivity analyses	Methods — Sensitivity Analysis: six analyses (Pearson correlations; polychoric–polyserial matrix; EBIC γ varied 0–0.5; fully subscale-level network; network comparison test, married vs. dating; 12-node network with global relationship quality removed)	14
**Results**			
13a	Report numbers of individuals at each stage of the study	Results — Participant Characteristics; Methods — Missing Data (476 completed; 472 analysed)	10, 15
13b	Give reasons for non-participation at each stage	Not applicable: the survey was anonymous and voluntary, so non-participation could not be recorded	—
13c	Consider use of a flow diagram	Not applicable: only one exclusion step (incomplete network data, n = 4)	—
14a	Give characteristics of study participants and information on exposures and confounders	Results — Participant Characteristics; Table 1	15–16
14b	Indicate the number of participants with missing data for each variable of interest	Methods — Missing Data; Results — Participant Characteristics	10, 15
15	Report numbers of outcome events or summary measures	Results — Table 2; Network Structure	17

16a	Give unadjusted estimates and, if applicable, adjusted estimates and their precision	Results — Network Structure (zero-order Spearman correlations and regularised partial correlations); Network Accuracy and Stability (bootstrap 95% CIs; CS-coefficients); Figures 1–3; Supplementary Tables S1–S2	17, 20–21
16b	Report category boundaries when continuous variables were categorised	Table 1 (age groups)	16
16c	If relevant, consider translating estimates of relative risk into absolute risk	Not applicable: no risk estimates were computed	—
17	Report other analyses done (subgroups, interactions, sensitivity analyses)	Results — Participant Characteristics (redundancy analysis); Network Structure (community-detection algorithms; edge-weight difference tests); Centrality Analysis (node predictability); Sensitivity Analysis (Pearson, polychoric–polyserial, γ variation, subscale-level network, network comparison test, 12-node network)	14–22
**Discussion**			
18	Summarise key results with reference to study objectives	Discussion, opening paragraph (three main findings stated against the study aims)	23
19	Discuss limitations, including sources of potential bias or imprecision	Discussion — Limitations (cross-sectional design; weak and only moderately stable bridge estimates; single-informant self-report; mixed node levels; unexamined moderators; redundancy and local dependence; non-invariance across relationship status; sample composition)	29
20	Give a cautious overall interpretation considering objectives, limitations, and other evidence	Discussion (interpretation of community structure, edge magnitudes, redundancy, and the married–dating structure difference); Implications	23–29
21	Discuss the generalisability (external validity) of the study results	Discussion — Limitations (non-probability sample; women over-represented; single national context)	29
**Other information**			
22	Give the source of funding and the role of the funders	Funding statement	32

Note. An Explanation and Elaboration article discusses each checklist item and is available together with the official checklist at https://www.strobe-statement.org. If the journal requires its own STROBE template, transfer the entries in the third and fourth columns to that form.

Supplementary Table S1 Zero-order Spearman correlations among the study composites

Variable	1	2	3	4	5
1. Partner phubbing	—				
2. Relationship quality	−0.33	—			
3. Intimacy	−0.31	0.87	—		
4. Passion	−0.27	0.83	0.86	—	
5. Commitment	−0.25	0.86	0.90	0.90	—

Notes: Spearman correlations; all |ρ| ≥ 0.25 significant at $p < 0.001$.

Supplementary Table S2 Node descriptions and standardized centrality and bridge centrality

Node	Description	Community	Strength	EI	Bridge str.	Bridge EI
Ph1	Uses phone during a shared meal	Phubbing	0.21	0.39	-0.76	0.38
Ph2	Keeps phone visible when together	Phubbing	-2.05	-1.76	-0.51	1.36
Ph3	Holds phone when I am beside them	Phubbing	0.06	0.29	-0.92	0.51
Ph4	Checks phone mid-conversation	Phubbing	-0.10	-0.32	0.64	-0.73
Ph5	Glances at phone while talking	Phubbing	0.23	-0.02	0.76	-0.82
Ph6	Uses phone during shared free time	Phubbing	0.61	0.89	-1.09	0.64
Ph7	Uses phone while talking (reverse-recoded)	Phubbing	-2.37	-2.28	-0.59	0.25
Ph8	Uses phone when out together	Phubbing	0.60	0.51	0.21	-0.39
Ph9	Uses phone when the conversation lulls	Phubbing	0.51	0.77	-0.87	0.74
Intimacy	Intimacy (Triangular Love Scale)	Relationship	0.81	0.36	1.45	-1.37
Passion	Passion (Triangular Love Scale)	Relationship	0.17	0.22	-0.24	0.12
Commitment	Commitment (Triangular Love Scale)	Relationship	1.16	1.50	-0.44	1.43
Quality	Global relationship quality	Relationship	0.17	-0.56	2.38	-2.11

Notes: Values are z-scores. EI = expected influence; Bridge str. = bridge strength; Bridge EI = bridge expected influence (one-step). n = 472.

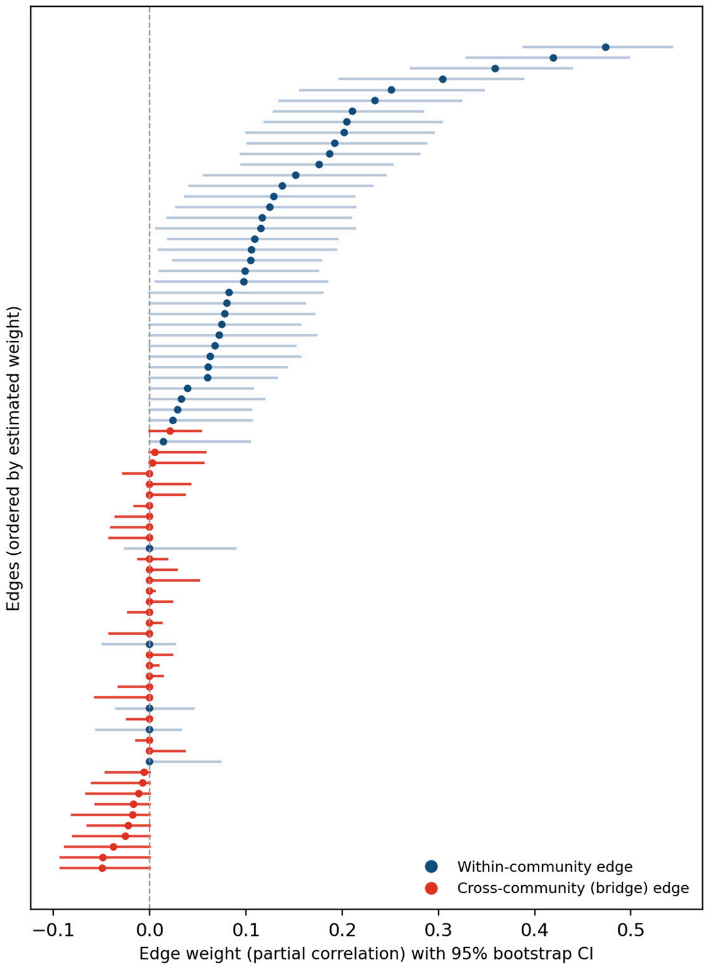

Supplementary Figure S1 Bootstrapped 95% confidence intervals for all edge weights, ordered by the sample estimate. Cross-community (bridge) edges are highlighted in red and cluster near zero; every bridge interval includes zero.

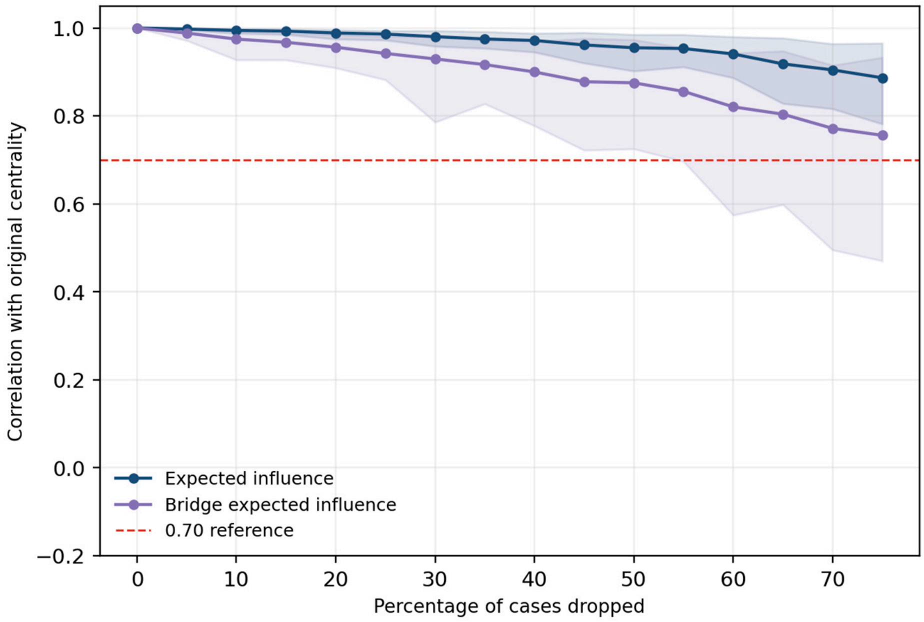

Supplementary Figure S2 Case-dropping bootstrap stability of expected-influence and bridge expected-influence centrality. Expected influence remained highly stable; bridge centrality declined earlier, indicating lower stability.

Supplementary Table S3 Item-level descriptive statistics for the nine partner-phubbing items.

Item	Description	Mean	SD	Range
Ph1	Uses phone during a shared meal	2.74	1.32	1–5
Ph2	Keeps phone visible when together	3.78	1.36	1–5
Ph3	Holds phone when I am beside them	2.23	1.23	1–5
Ph4	Checks phone mid-conversation	2.73	1.33	1–5
Ph5	Glances at phone while talking	2.04	1.23	1–5
Ph6	Uses phone during shared free time	2.82	1.32	1–5
Ph7	Uses phone while talking (reverse-recoded)	3.19	1.39	1–5
Ph8	Uses phone when out together	2.98	1.28	1–5
Ph9	Uses phone when the conversation lulls	2.84	1.37	1–5

Notes: n = 476 completers. Items rated 1–5 (higher = more perceived phubbing); Ph7 reverse-recoded. Inter-item Spearman correlations ranged from 0.10 to 0.63.
